# Solasonine Suppresses the Proliferation of Acute Monocytic Leukemia Through the Activation of the AMPK/FOXO3A Axis

**DOI:** 10.3389/fonc.2020.614067

**Published:** 2021-01-29

**Authors:** Hong Zhang, Fang Tian, Pengjun Jiang, Shushu Qian, Xingbin Dai, Bangyun Ma, Mengya Wang, Huibo Dai, Xiaocao Sha, Zhongfa Yang, Xuejun Zhu, Xuemei Sun

**Affiliations:** ^1^Jiangsu Province Hospital of Chinese Medicine, Affiliated Hospital of Nanjing University of Chinese Medicine, Nanjing, China; ^2^Institute of Clinical Medicine, Weifang Medical University, Weifang, China

**Keywords:** solasonine, *Solanum nigrum L*., acute monocytic leukemia, AMPK, FOXO3A, AMPK/FOXO3A, Zebrafish Xenograft

## Abstract

Solasonine, the main active ingredient of *Solanum nigrum L*., has been reported to exert extensive antitumor activity. However, the antitumor effects in acute monocytic leukemia and the exact mechanisms involved are unknown. In this study, we investigated the role of solasonine on inhibiting the progression of acute monocytic leukemia. Our findings showed that solasonine inhibited the proliferation of acute monocytic leukemic cell lines (THP-1 and MV4-11) *in vitro*. Solasonine promoted apoptosis and induced cell cycle arrest in the G2/M phase. Analysis of RNA-seq data suggested that solasonine correlated with increased expression of genes in the AMPK/FOXO3A pathway. Inhibition of AMPK with compound C followed by treatment with solasonine showed that solasonine reduced apoptosis, caused less cell cycle arrest, and inactivated the AMPK/FOXO3A axis in THP-1 and MV4-11 cells. Solasonine also inhibited tumor growth by the activation of the AMPK/FOXO3A axis. In conclusion, solasonine inhibited the progress of acute monocytic leukemia *in vitro* and *in vivo* and triggered the apoptosis and cell cycle arrest in the G2/M phase by upregulating the AMPK/FOXO3A pathway.

## Introduction

Acute myeloid leukemia (AML) is a hematopoietic malignancy derived from hematopoietic stem cells with high clinical and biological heterogeneity. Acute monocytic leukemia or AML-M5 is a subtype of AML defined by the French American-British (FAB) classification. Acute monocytic leukemia is characterized by a large number of myeloid derived monoblasts, promonocytes, and monocytes in the bone marrow and peripheral blood. Based on the morphological analysis, acute monocytic leukemia is classified as M5a or M5b, distinguished by the relative proportion of monoblasts and promonocytes (1). Clinically, AML-M5 is manifested by hyperleukocytosis (2), extramedullary infiltration (3), and coagulation abnormalities (4). Cytogenetic changes including gene mutations and chromosomal translocations occur frequently in this disease, such as MLL gene translocations on chromosome 11q23 (5), DNMT3A (6), and NPM1 (7) gene mutations. Considering the poor prognosis and unsatisfactory efficacy of the therapies, it is urgent to explore new therapeutic strategies to treat this disease.

*Solanum nigrum L*., a traditional Chinese medicine (TCM) herb from the Solanaceae plant family, is distributed all over the world and has been used for its whole grass and fruit. Previous studies indicated that *Solanum nigrum L*. had various activities such as inhibiting hepatic (8), cervical (9), breast (10), prostate (11), and colorectal cancers (12), the formation of hepatic fibrosis (13) as well as hepatoprotective effects (14). Solasonine is the main active ingredient of *Solanum nigrum L*., with extensive antitumor activity (15). The antitumor mechanism of solasonine included: inhibition of the growth of tumor cell lines by altering the membrane structure and reducing its fluidity (16), inducing apoptosis of tumor cells and inhibition of tumor cell transcription (17). However, the effects of solasonine on acute monocytic leukemic cells have not been extensively studied.

The family of FOX transcription factors were first identified in 1989 because they shared a common conserved DNA binding site. The FOX family has 19 subfamilies, from FOXA to FOXS. FOXO3A is a part of the FOXO subfamily. FOXO3A plays an important role in the response to cellular oxidative stress, energy metabolism regulation, cell cycle regulation, apoptosis, and autophagy (18). Post-translational modifications, including phosphorylation, ubiquitination, and acetylation are regulated FOXO3A, which in turn regulates the intracellular localization and the expressions of downstream target genes (19). AMPK also regulates energy metabolism by controlling biochemical reactions of glucose and lipid metabolism. The tumor suppressor kinase LKB1 activates the downstream AMPK, and then regulates energy metabolism and control of cell growth (20). The activated AMPK can induce FOXO3A nuclear accumulation and in turn biological functions included autophagy, cell cycle arrest, and cell death (21). In breast and hepatocellular cancer, AMPK-activated FOXO3A induced cell cycle arrest and apoptosis (22, 23). Therefore, it is necessary to further identify the exact role played by the AMPK/FOXO3A axis in acute monocytic leukemia. In the present study, we aimed to investigate the role of solasonine on inhibiting the progression of acute monocytic leukemia and the underlying mechanism.

## Materials and Methods

### Cell Culture and Compounds

The human leukemic cell lines THP-1, MV4-11, NB-4, HL-60, HEL, Raji, and Jurkat were purchased from the ATCC (ATCC, USA). All cell lines were cultured in Roswell Park Memorial Institute 1640 (RPMI 1640) (Gibco, USA) or Iscoves Modified Dulbecco’s Medium (IMDM) (HyClone, USA) supplemented with 10% fetal bovine serum (Evergreen Company, China), 100 U/ml ampicillin, and 100 g/ml streptomycin (Life Technologies) at 37°C in a 5% CO_2_ incubator. Solasonine (HPLC≥ 98%) was purchased from Shanghai Yuanye Bio-Technology Co., Ltd (Shanghai, China) and Ara-C was obtained from MedChemExpress LLC (Shanghai, China), both were dissolved in dimethyl-sulfoxide (DMSO) to obtain a 100 mmol/L stock solution and stored at −20°C and then diluted in working concentrations before use. Compound C was purchased from AbMole (USA) and dissolved in PBS to 10 mmol/L.

### CCK‐8 Assay

The CCK-8 assay was used to evaluate the proliferation and cytotoxic activity of solasonine in THP-1, MV4-11, NB-4, HL-60, HEL, Raji, and Jurkat cell lines. In brief, 5×10^4^/well cells were seeded in 96-well plates with 100 μl of their respective media and treated with 100 μl of different concentrations of solasonine, and then cultured for 24 or 48 h. CCK-8 solution (10 μl) was then added to each well and the cells were incubated during the last 4 h. There were six replicates for each concentration and the experiment was repeated three times. The optical density (OD450) absorption was measured using an automatic microplate reader (BioTek Instruments, VT, United States). The cell inhibition rate was calculated as =1– (absorbance of test sample/absorbance of control sample) × 100%.

### Flow Cytometry Analysis

Flow cytometry analysis was used to detect the cell apoptosis and cell cycle in THP-1 and MV4-11 cell lines. In brief, 1×10^5^ cells/well suspended in a 2 ml volume of culture media in 24-well plates and treated with different doses of solasonine, 2 μM compound C, and 8 μM solasonine+2 μM compound C for 24 h. Subsequently, the cells were collected, washed with 1 ml ice cold-PBS, and incubated with 500 μl ice-binding buffer (1×) and 5 μl of Annexin V-FITC and 10 μl of propidium iodide (PI) in the dark for 15 min according to the manufacturer’s protocol of the apoptosis kit (MultiSciences, Hangzhou, China). Finally, flow cytometry (Beckman Coulter, USA) was used to detect apoptosis rates. For the cell cycle analysis, the cells were treated the same as the cells tested for apoptosis and then cells were collected, washed with PBS, and fixed with 70% cold ethanol for 30 min. Fixed cells were washed with PBS and incubated with 1 ml DNA staining solution and 10 μl permeabilization solution in the dark for 30 min according to the protocol of the Cell Cycle Kit (MultiSciences, Hangzhou, China). Finally, flow cytometry was used to detect the percentages of cells in different stages during the cell cycle. FlowJo (BD, United States) software was used to analyze apoptosis, and Kaluza (Beckman Coulter, USA) software was used to analyze the cell cycle.

### Immunoblotting Analysis

THP-1 and MV4-11 cells (1×10^5^/ml) suspended in 6 ml culture media were seeded in a 6-well culture plate and treated with different concentration of solasonine, 2 μM compound C, or 8 μM solasonine+2 μM compound C for 24 h. Cells were lysed with RIPA buffer (Beyotime, China) containing inhibitor cocktail (MedChem Express, USA). The nuclear and cytosolic protein lysates were lysed according the protocol specified by the Nuclear and Cytoplasmic Protein Extraction Kit (Beyotime, China). The mouse tumor tissue proteins were extracted and ground after addition of RIPA lysis buffer, followed by centrifuging at 20,000*g for 20 min at 4°C. The supernatant was collected, and its concentration was determined using the BCA Protein Assay Kit (Beyotime, China). Sodium dodecyl sulfate polyacrylamide gel electrophoresis (SDS-PAGE) separated the proteins, which were then transferred to polyvinylidene difluoride (PVDF) membrane (Millipore, Billerica, USA). After blocking with 5% skimmed milk, the PVDF membrane was incubated with the following primary antibodies for 12 h: PARP (Affinity, United States, 1:1,000 dilution), caspase 9 (Cell Signaling Technology, United States, 1:1,000), cleaved caspase 9 (Cell Signaling Technology, United States, 1:1,000), caspase 3 (Proteintech, United States, 1:1,000), cleaved caspase 3 (Cell Signaling Technology, United States, 1:1,000), Bax (Cell Signaling Technology, United States, 1:1,000), Bcl-2 (Cell Signaling Technology, United States, 1:1,000), cyclin B1 (Proteintech, United States, 1:1,000), CDK1 (Proteintech, United States, 1:1,000), phosphorylated (P)-CDK1 (Affinity, United States, 1:1,000 dilution), AMPK (Proteintech, United States, 1:1,000 dilution), phosphorylated (P)-AMPK(Affinity, United States, 1:1,000 dilution), FOXO3A (Proteintech, United States, 1:1,000 dilution), β-actin (Affinity, United States, 1:5,000 dilution), and histone H3 (Affinity, United States, 1:1,000 dilution). The PVDF membrane was then incubated with a goat anti-rabbit IgG (Affinity, United States, 1:5,000) secondary antibody for 2 h. Finally, images were visualized by ChemiDoc XRS+ (Bio-Rad, United States) and analyzed by Image Lab, version 5.2.1(Bio-Rad Laboratories, Inc., United States).

### Real-Time Quantitative PCR Analysis

THP-1 and MV4-11 cells (1×10^5^/ml) suspended in 6 ml were seeded in a 6-well culture plate and treated with different concentration of solasonine (0, 6, 8, or 10 μM) for 24 h. Total RNA was isolated using TRIzol (Ambion, USA) from THP-1 and MV4-11 cell lines and complementary DNA (cDNA) was synthesized using the 5×All-In-One RT MasterMix Kit (ABM, China) under the following cycling conditions: 42°C for 15 min and 85°C for 5 min. Real-time quantitative PCR (RT-qPCR) was performed with a 7500 RT-PCR system (Applied Biosystems, USA). PCR reactions included EvaGreen 2×qPCR MasterMix (ABM, China), forward primers, reverse primers (Sangon Biotech, China), cDNA, and DEPC H2O (Beyotime, China). Procedures were set as 40 cycles of 95°C for 15 s, and 60°C for 1 min. The 2^−ΔΔCT^ method was used to analyze RNA expression levels. The RNA expression level was calculated as =2^−ΔΔ (the target CT of test sample-the ref CT of test sample)-(the target CT of control sample-the ref CT of control sample)^. The following primer sequences were used: actin: forward, 5′-TCACCCACACTGTGCCCATCTACGA-3′, reverse, 5′-CAGCGGAACCGCTCATTGCCAATGG-3′; AMPK: forward, 5′- CCTCTCGAAAGGTGGACAGC-3′, reverse, 5′- CTCCTGGTAGGAGAACGGGA-3′; FOXO3A: forward, 5′- GCAAAGCAGACCCTCAAACTG-3′, reverse, 5′- GCGTGGGATTCACAAAGGTG-3′.

### Immunofluorescence Staining

THP-1 and MV4-11 cells (1×10^5^/ml) suspended in 6 ml culture media were seeded in 6-well plates and then treated with 8 μM solasonine and cultured for 24 h. Cells were collected into a 1.5 ml Eppendorf tube and washed with PBS, and subsequently, fixed with 1% paraformaldehyde for 20 min, permeabilized with 0.1% Triton X-100 for 1 h, and blocked with 1% BSA for 1 h. Fixed cells were incubated with the anti-FOXO3A antibody (Proteintech, United States, 1:200) overnight at 4°C. After a PBS wash, cells were incubated with a goat anti-IgG/Cy-3 secondary antibody (Wuhan Good bio Technology Co., Ltd, China, 1:500) for 1 h at room temperature, and then stained with DAPI (Beyotime, China) for 30 min. Finally, cell images were captured using a camera equipped a Laser Scanning Confocal Microscope (Zeiss 710, Germany).

### TUNEL Assay

TUNEL assays were used to detect apoptosis induced by solasonine on THP-1 and MV4-11 cell lines. In brief, THP-1 and MV4-11 cells (3×10^5^/ml) were exposed to 8 μM solasonine for 24 h. Cells were collected into 1.5 ml Eppendorf tubes, fixed with 1% paraformaldehyde for 30 min, and then permeabilized with 0.1% Triton X-100 for 5 min. Cells were then resuspended in 100 μl of 1×equilibration Buffer for 5 min, and then incubated with BrightRed Labeling liquid TdT for 1 h in the dark. Cells were then stained with DAPI for 30 min according to the TUNEL Assay Kit (Vazyme, China) protocol. Cell images were captured with a fluorescent microscopy (Nikon80i, Nikon, Tokyo, Japan).

### Mouse Xenograft Studies

Twenty female BALB/c nude mice at 5–6 weeks of age were housed under a pathogen free (SPF) environment. A total of 5×10^6^ THP-1 cells were subsequently injected into right forelimb of each mice. When the tumor volume grew to 100 mm^3^, the mice were randomly separated into four groups (four mice in each group): group 1 were treated with normal saline as the NS control group; group 2 mice were treated with a low dose of solasonine (4 mg/kg); group 3 mice were treated with an intermediate dose of solasonine (8 mg/kg); and group four received a high dose of solasonine (16 mg/kg). The dosages were firstly chosen based upon *in vitro* cell lines IC50 data. The maximal dosage was determined by administering 16 and 20 mg/kg solasonine to healthy young mice. The mice receiving dosage 16 mg/kg were relatively unaffected, while the mice receiving 20 mg/kg demonstrated signs of detrimental condition. Solasonine was dissolved in DMSO to 100 mmol/L stock solution stored at −20°C and then diluted in working concentration before use. All the mice received intraperitoneal injections once a day for 14 days, the body weight and the tumor size were measured once every 2 days for 14 days (24, 25). All mice were subjected to euthanasia on the 14th day and then tumor tissues were separated. Animal experiments were approved by the Animal Ethics committee of the Affiliated Hospital of Nanjing University of Traditional Chinese Medicine (NO: 2019DW-21-02). The tumor volume was calculated as follows: V (tumor volume, mm^3^) = 0.5 × a (tumor length, mm) × b (tumor width, mm)^2^.

### Zebrafish Xenograft Studies

Zebrafish were purchased from the Model Animal Research Center of Nanjing University and maintained at 28°C in a circulating water system. Zebrafish embryos were obtained when adult males and females zebrafish were housed together. Embryos were collected and placed at 28°C in petri dishes containing embryo culture medium. In brief, zebrafish embryos at 24 h post fertilization (24 hpf) were dechorionated with 1 mg/ml of pronase (Sigma-Aldrich, USA), then 2 mM 1-phenyl 2-thiourea (PTU) (Sigma-Aldrich, USA) was added to the embryo culture medium and incubated at 28°C. At 48 hpf, embryos were anesthetized with 0.0003% tricaine (Sigma-Aldrich, USA), positioned on a agar plate and approximately 200-300 CM-DiI THP-1 cells were injected into the yolk sac per embryo using a microinjector (Nanoliter 2010, USA), while under observation by stereoscope (Nikon, Japan). At 72 hpf, 120 zebrafish embryos xenografts having the same fluorescence intensity were randomly divided into three groups (40 embryos per group): the experimental group was exposed to 10 μM solasonine, the positive drug treatment group received four μM Ara-C, and the untreated negative control group. All groups were cultured in 6-well plates containing embryos in 5 ml in culture medium for a treatment period of 4 days in a incubator at 28°C. Twenty embryos of each group were used to study the inhibitory effect of the drugs on tumor cells, and the other twenty were used to observe the survival of zebrafish. The growth and metastasis of the tumor were observed using a fluorescence inverted microscope (Nikon Inc., Tokyo, Japan). The three zebrafish groups were sacrificed and digested with collagenase to single cell suspensions at 4 dpt and the number of CM-DiI stained cells untreated with drugs at 0 dpt were set a baseline number of 4 dpt.

In order to ensure the maximum safe doses of drugs, 100 zebrafish embryos at 72 hpf were treated respectively with concentrations of solasonine (0, 5, 10, 15, 20, or 25 μM) and Ara-C (0, 1, 2, 4, 8, or 16μM) for 4 days in 5 ml embryos culture medium at 28°C in an incubator. Fresh embryo culture medium with different drug concentrations was changed every 24 h for 4 treatment days. The survival rate of zebrafish embryos under different dosages was calculated to determine the maximum safe dose of solasonine and the positive control drug.

### Immunohistochemical Staining

Tumor tissues were fixed in 4% paraformaldehyde for 24 h and were dehydrated through a serial concentrations of alcohol washes, embedded in paraffin and cut into 5-mm thick sections. Before immunostaining, the tissues were dewaxed in xylene and blocked with 10% sheep serum for 1 h at 37°C. The tissues sections were incubated with the KI-67, Bax, Bcl-2, cyclin B1, AMPK, or FOXO3A primary antibodies (Proteintech, United States, 1:200 dilution) overnight. The sections were then incubated with HRP labeled goat anti-rabbit IgG secondary antibody (Servicebio, Wuhan, China) for 1 h. Finally, the sections were stained with hematoxylin and images were captured by fluorescent microscopy using a Pannoramic 250 Flash III.

### Next-Generation RNA Sequencing

THP-1 cells (3×10^5^/ml) were seeded into 25 cm^2^ culture flasks and treated with solasonine (0 or 8 mM) for 24 h. The experiment was repeated three times with independent cell samples. Total RNA was isolated using TRIzol-based method (Ambion, USA) from THP-1 cells, and the extracted RNA was sent to Sangon Biotech Co (Shanghai, China) for sequencing under Illumina HiSeq platform. Pair-end reads were first trimmed (Trimmomatic) to remove the sequencing adapter sequence and were then filtered (FastQC) out low quality reads. Cleaned high quality sequence reads were then aligned (HISAT2) onto human genome reference with annotation. Mapped sequence reads were quantified at gene-level with StringTie software. Differential gene expression analysis between the control and treatment groups was performed with the R package DESeq2. Genes with q value<0.05 and the absolute fold change >2 were considered as significantly differential expressed genes (DEGs). Associated gene expression analysis was performed with the weighted gene co-expression network analysis (WGCNA) model. Protein interaction network was constructed upon STRING network database. Gene ontology (GO) enrichment was performed with the topGO tool. Gene pathway enrichment was performed based upon the Kyoto Encyclopedia of Genes and Genomes (KEGG) database.

### Statistical Analysis

SPSS version 26 and GraphPad Prism 6.0 software were used to analyze data which is expressed as mean ± SD. Two groups were compared with Student’s t test, and multiple groups were compared with One-way analysis of variance (ANOVA). P-values < 0.05 were considered to indicate a statistically significant difference.

## Results

### Solasonine Inhibited Cells Proliferation

**Figure 1:**
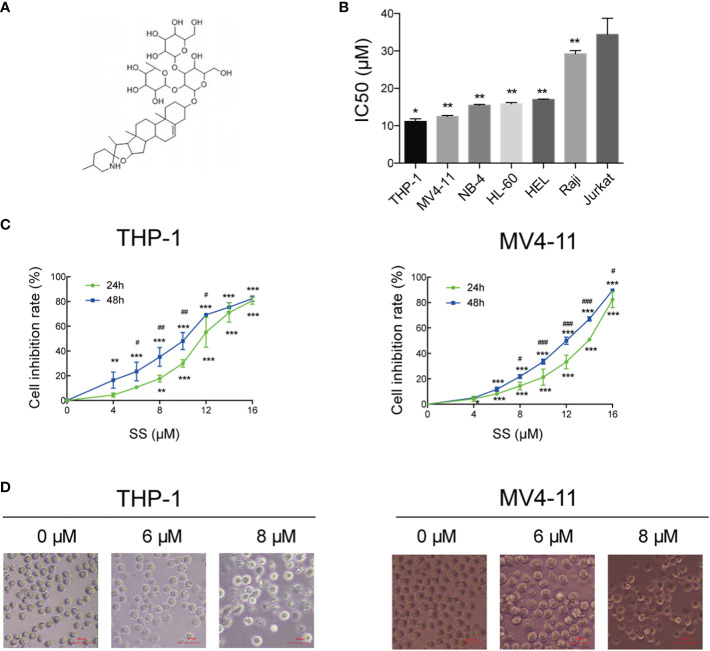
Solasonine inhibited cell proliferation. **(A)** Chemical structures of solasonine. **(B)** IC50 value of solasonine in THP-1, MV4-11, NB-4, HL-60, HEL, Raji, and Jurkat cell lines. **(C)** Inhibition rate of THP-1 and MV4-11cells at different time points and concentrations. **(D)** Morphological changes of THP-1 and MV4-11cells induced by solasonine. (*considered a statistical difference compared the different concentrations to control group at the same time point, ^#^considered a statistical difference compared 48 h to 24 h at the same concentration. ^*#^P < 0.05, ^**##^P < 0.01, ^***###^P < 0.001).

**Figure 1A** shows the chemical structures of solasonine. We performed the CCK-8 assay using seven leukemia cell lines (THP-1, MV4-11, NB-4, HL-60, HEL, Raji, and Jurkat) to evaluate the proliferation and cytotoxic activity of solasonine. The results showed that solasonine exerted different levels of inhibitory effects on leukemia cell lines, among which the inhibitory effect on AML cell lines (THP-1, MV4-11, NB-4, HL-60, HEL) were stronger and in acute lymphocytic leukemia (ALL) cell lines (Raji and Jurkat) were weaker. Among the AML cell lines, the M5 subtype (AML-M5) cell lines THP-1 and MV4-11 were the most sensitive with a calculated IC50 of 11.19 and 12.50 μM, respectively; the AML-M3 cell lines HL-60 and NB-4 had an IC50 of 15.875 and 15.456 μM, respectively; and the AML-M6 cell line HEL, the IC50 was 17 μM (**Figure 1B**). Therefore, we chose THP-1 and MV4-11 cell lines for further test. Through this process, we found that increasing concentrations of solasonine inhibited the proliferation of THP-1 and MV4-11 cell lines in both 24 and 48 h (**Figure 1C**); We also observed morphological changes of the two cell lines: the solasonine treated cells were fewer in number than the control group cells, and with increasing concentrations of solasonine, a certain percentage of apoptosis-specific changes occurred in both cell lines, such as nuclear shrinkage and nuclear fragmentation (**Figure 1D**).

### Solasonine Promoted Apoptosis

**Figure 2:**
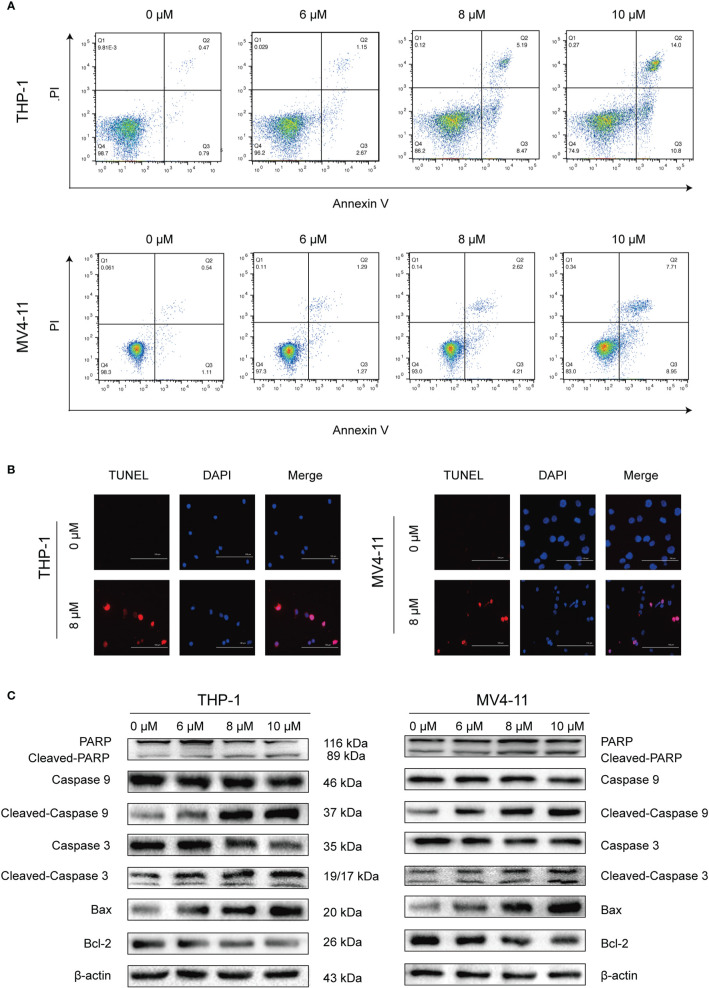
Solasonine promoted acute monocytic leukemic cells apoptosis. **(A)** The flow cytometry analysis indicated that the apoptosis rate increased with the increase of solasonine concentration in THP-1 and MV4-11 cell lines. **(B)** TUNEL assay detected apoptosis of DNA degradation in solasonine treatment. **(C)** Protein levels related to apoptosis conducted by immunoblotting analyses in THP-1 and MV4-11cells.

After determining that solasonine could inhibit cell proliferation using the CCK-8 assay, we decided to further investigate the functional activity of solasonine on acute monocytic leukemic cell lines by conducting flow cytometry, immunoblotting, and TUNEL assays. Firstly, the flow cytometry analysis indicated that the apoptosis rate increased with the increase of solasonine concentration in THP-1 and MV4-11 cell lines (**Figure 2A** and **Supplementary Figure 1A**). Secondly, the TUNEL assay further detected apoptosis of DNA degradation in solasonine treatment (**Figure 2B**). Lastly, to confirm the relationship between solasonine and apoptosis-associated protein, immunoblotting was performed and it revealed that solasonine promoted the expression of pro-apoptosis protein (Bax) and apoptosis-associated proteins (cleaved-caspase3 and cleaved-caspase9), and inhibited the expression of the anti-apoptotic protein Bcl-2 (**Figure 2C** and **Supplementary Figure 1B**). Thus, solasonine had a positive effect on promoting apoptosis of acute monocytic leukemic cells.

### Solasonine Induced Cell Cycle Arrest

**Figure 3:**
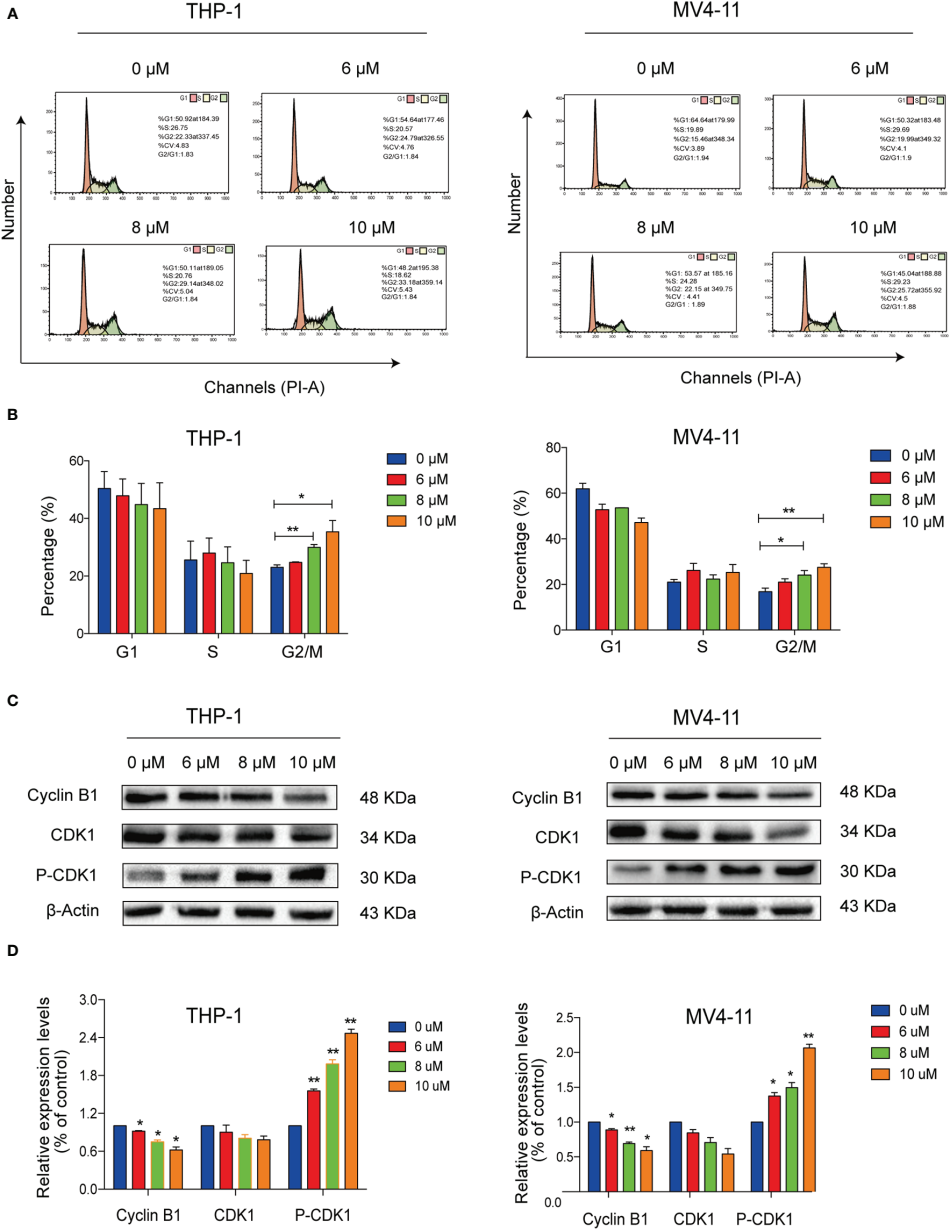
Solasonine induced acute monocytic leukemic cell cycle arrest in the G2/M phase. **(A)** Flow cytometric analysis showed that solasonine increased the proportion of cells in the G2/M phase in THP-1 and MV4-11cells. **(B)** Bar chart showed the percentage of cells in different cell cycle phase. **(C)** Immunoblotting analysis reported that solasonine upregulated the expression of P-CDK1 and downregulated Cyclin B1 in THP-1 and MV4-11 cells. **(D)** Bar chart showed the relative protein expression level of cells in the G2/M phase. (*considered a statistical difference compared to the control group, *P < 0.05, **P < 0.01).

We conducted flow cytometry analyses to determine the effects of solasonine on cell cycle progression on THP-1 and MV4-11 cell lines. Our results indicated that solasonine increased the proportion of cells in the G2/M phase (**Figures 3A, B**). Additionally, immunoblotting analyses further indicated that solasonine had an effect on the G2/M phase- related protein-it reduced the expression of Cyclin B1 and increase the phosphorylation of CDK1(P-CDK1) (**Figures 3C, D**). These results demonstrated that solasonine suppressed the cell cycle progression and arrested cells in G2/M phase.

### Solasonine Activated the AMPK/FOXO3A Signaling Pathway

**Figure 4:**
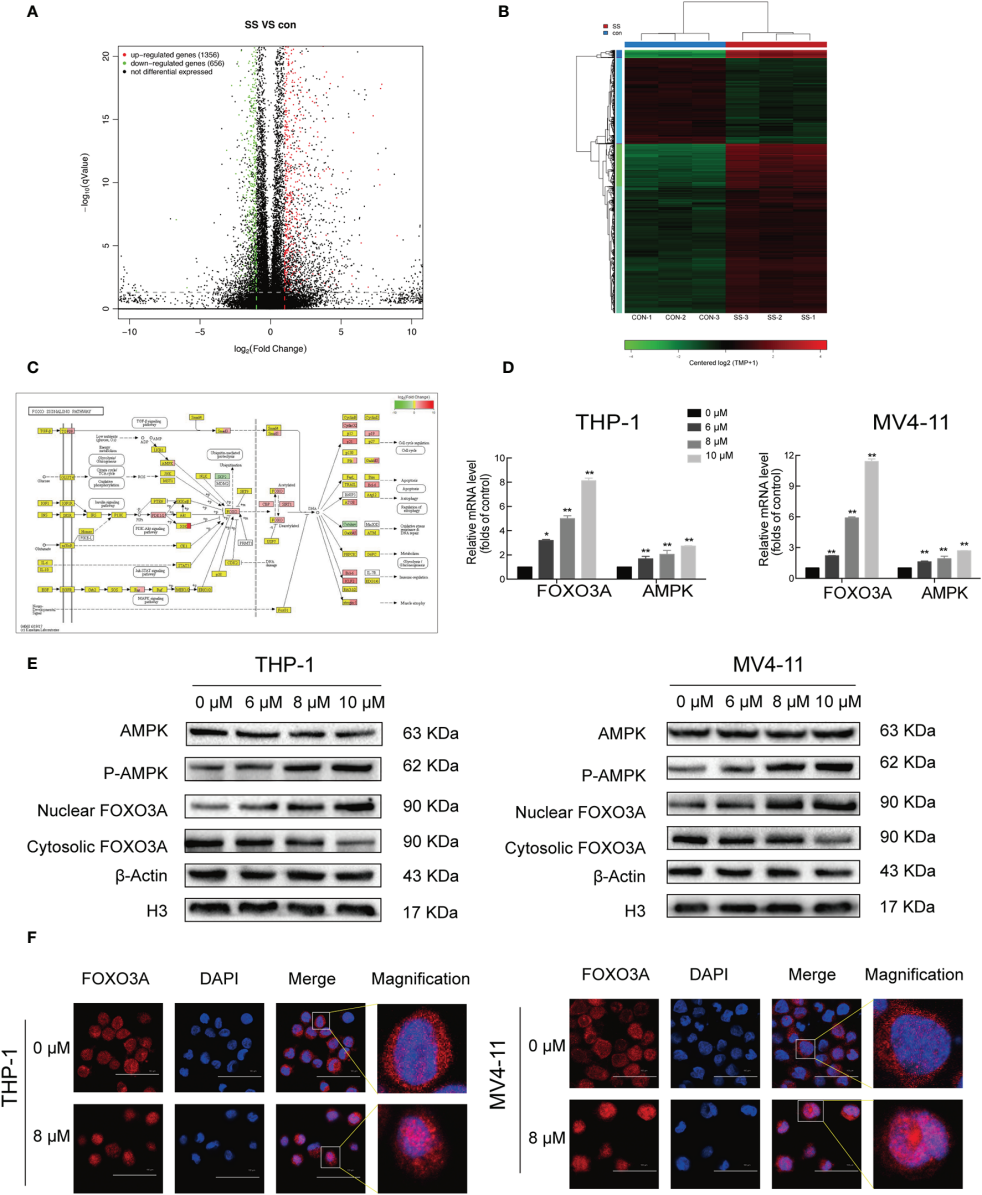
Solasonine activated AMPK/FOXO3A axis. **(A)** The next-gen RNA sequencing of volcano plot with 1,912 DEGs, including 1,356 upregulated and 656 downregulated gene from solasonine-treated and untreated THP-1. **(B)** The next-gen RNA sequencing of clustering heat map. **(C)** The next-gen RNA sequencing found that a subset of DEGs were specifically enriched in the FOXO signaling pathway. **(D)** Relative RNA expression of AMPK and FOXO3A in THP-1 and MV4-11cells by RT-qPCR. **(E)** Protein levels by immunoblotting analysis in THP-1 and MV4-11cells indicated that solasonine upregulated the expression of P-AMPK and caused nuclear translocation of FOXO3A. **(F)** Immunofluorescence staining showed that solasonine induced nuclear translocation of FOXO3A. (*considered a statistical difference compared to the control group, *P < 0.05, **P < 0.01).

Given the results showing the anti-proliferative, pro-apoptotic, and cell cycle arrest effects induced by solasonine, we next sought to explore the underlying mechanisms through next generation RNA sequencing. RNASeq data have been deposited in SRA database with the number of PRJNA669277. A total of 1912 differentially expressed genes (DEGs), including 1,356 upregulated and 656 downregulated genes were obtained from the comparison between solasonine-treated and untreated THP-1 cells (**Figure 4A**). These genes were clearly clustered into two separate groups that coincided with solasonine-treated and untreated THP-1 cells (**Figure 4B**). We next classified the DEGs by performing a GO analysis and found that most DEGs were enriched in the cellular processes of apoptosis and the cell cycle. We also applied KEGG pathway enrichment analysis on the 1,912 DEGs and determined that a subset of DEGs was specifically enriched in the FOXO signaling pathway (**Figure 4C**). From the FOXO signaling pathway we found that the AMPK gene lies upstream of the FOXO gene. To evaluate the expression of FOXO3A and AMPK genes, we performed RT-qPCR analysis on messenger RNA (mRNA) isolated from solasonine treated THP-1 and MV4-11 cells. Solasonine increased the expression of AMPK and FOXO3A genes in both cell lines (**Figure 4D**). We also found, through immunoblotting analyses, that solasonine increased the expression of P-AMPK and caused nuclear translocation of FOXO3A in THP-1 and MV4-11 cell lines (**Figure 4E** and **Supplementary Figure 2**). Immunocytochemistry staining confirmed the nuclear translocation of FOXO3A after solasonine treatment (**Figure 4F**). Taken together, these results demonstrated that solasonine activated FOXO signaling by inducing the expression of its upstream AMPK and caused the nuclear translocation of FOXO3A.

### AMPK/FOXO3A Axis Was Required for Solasonine-Induced Cell Cycle Arrest and Apoptosis in Acute Monocytic Leukemic Cell Lines

**Figure 5:**
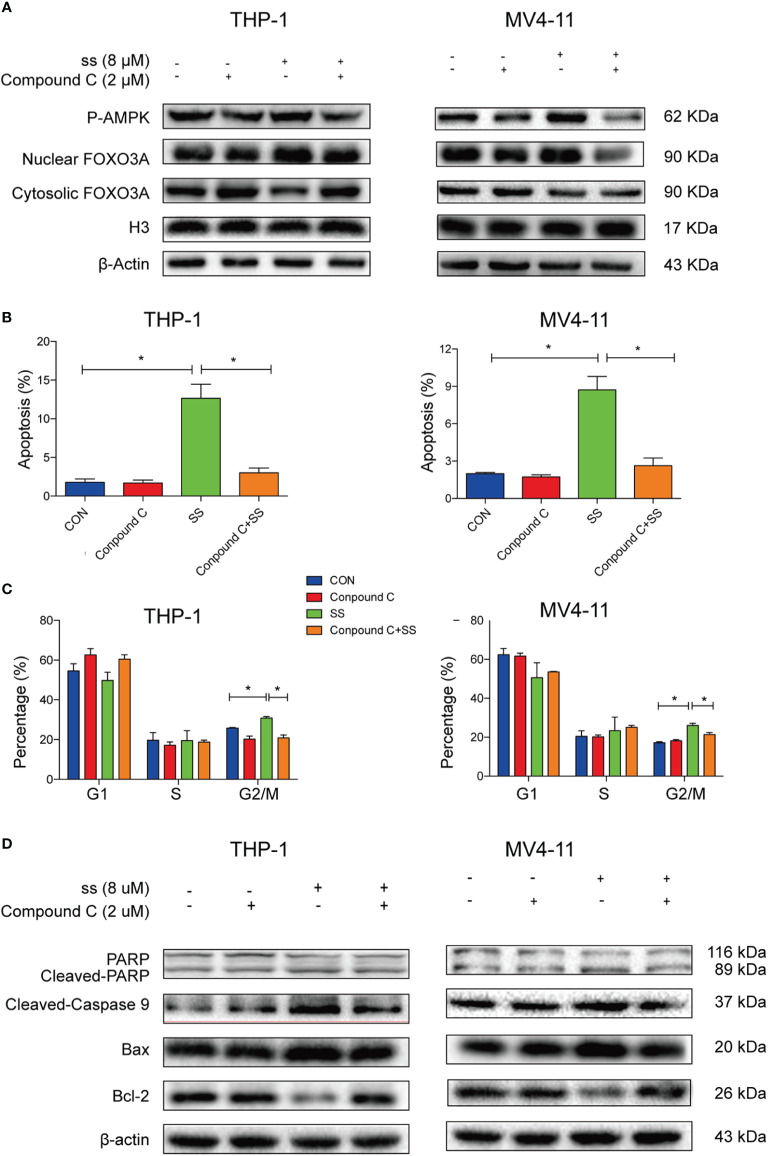
AMPK/FOXO3A signaling was required for solasonine-induced cell cycle arrest and apoptosis in acute monocytic leukemic cell lines. **(A)** Immunoblotting analysis showed that inhibition of AMPK activity by compound C prevented the solasonine-induced nuclear translocation of FOXO3A. **(B, C)** Bar chart showed that compound C decreased the solasonine induced apoptosis **(B)** and restored the cell cycle arrest in the G2/M phase **(C)** in THP-1 and MV4-11cells through Flow cytometry analysis. **(D)** Immunoblotting analysis showed that compound C decreased the solasonine-induced apoptosis related protein in THP-1 and MV4-11cells. (CON represented cells not treated solasonine and compound C; *P < 0.05).

To further confirm whether AMPK-FOXO3A axis is required to induced cell cycle arrest and apoptosis. We treated the cells with compound C, an inhibitor of AMPK. We observed that the compound C prevented the solasonine-induced nuclear translocation of FOXO3A in THP-1 and MV4-11 cell lines (**Figure 5A**). Meanwhile, we examined the effects of compound C on the rate of solasonine-induced apoptosis and cell cycle arrest in these cell lines. As show in **Figures 5B, C** and **Supplementary Figures 3A–C**, solasonine-induced apoptosis and cell cycle arrest in the G2/M phase was largely restored by the inhibitor of AMPK. Finally, the compound C treatment also decreased the expression of pro-apoptosis protein (Bax) and apoptosis-associated proteins (cleaved-caspase3, cleaved-caspase9, cleaved-PARP), and increased the expression of the anti-apoptotic protein Bcl-2 (**Figure 5D** and **Supplementary Figure 3D**). Thus, our findings showed that AMPK/FOXO3A signaling was molecular mechanisms involved in solasonine-induced apoptosis and cell cycle arrest in THP-1 and MV4-11 cells.

### Solasonine Demonstrated Its Anti-Tumor Activity in the Mouse Xenograft Model

**Figure 6:**
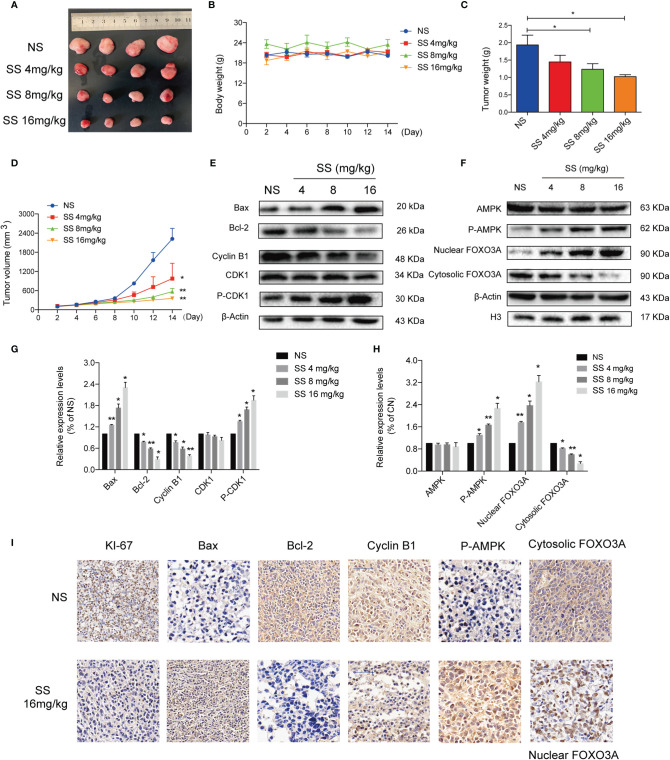
Solasonine demonstrated its anti-tumor activity in the mouse xenograft model. **(A)** Measurement of tumor size. **(B)** Body weight of xenograft mouse model indicated that there was no obvious difference. **(C, D)** The weight and size of tumor tissue declined with increases of the solasonine concentration. **(E)** Solasonine promoted apoptosis and cell cycle arrest showed by Immunoblotting analysis *in vivo*. **(F)** Solasonine increased the P-AMPK and induced FOXO3A nuclear translocation in tumor sections. **(G, H)** Bar chart showed the relative protein expression level related apoptosis, cell cycle, and AMPK/FOXO3A signaling *in vivo*. **(I)** Immunohistochemical (IHC) analysis showed that solasonine induced downregulation of KI-67, Bcl-2, and cyclin B1 and upregulated of Bax and P-AMPK. Furthermore, solasonine induced FOXO3A nuclear translocation *in vivo*. (NS represented cells treated normal saline, * considered a statistical difference compared to the NS group, *P < 0.05, **P < 0.01).

A xenograft mouse model was established to investigate whether solasonine could inhibit tumor growth *in vivo*. As shown in **Figures 6A–D**, the weight and size of tumor tissue decreased with increasing concentrations of solasonine treatment, while there were no obvious changes in total mouse body weight across the four groups. Immunoblotting analyses indicated that solasonine upregulated the expression of Bax and P-CDK1 and downregulated Bcl-2 and Cyclin B1 expression in mouse acute monocytic leukemic tissues (**Figures 6E, G**). Meanwhile, solasonine increased the P-AMPK and induced FOXO3A nuclear translocation in tumor sections (**Figures 6F, H**). Using IHC staining, we observed fewer KI67 positively stained cells in solasonine-treated mice, suggesting that solasonine inhibited tumor growth by reducing cell proliferation. We also observed that the expression of Bax and P-AMPK increased, while that of Bcl-2 and Cyclin B1 were decreased, and that FOXO3A nuclear translocation increased (**Figure 6I**). Overall, these results suggested that solasonine inhibited tumor growth *via* the activation of the AMPK/FOXO3A axis.

### Solasonine Inhibited Tumor Growth and Metastasis in the Zebrafish Xenograft Model

**Figure 7:**
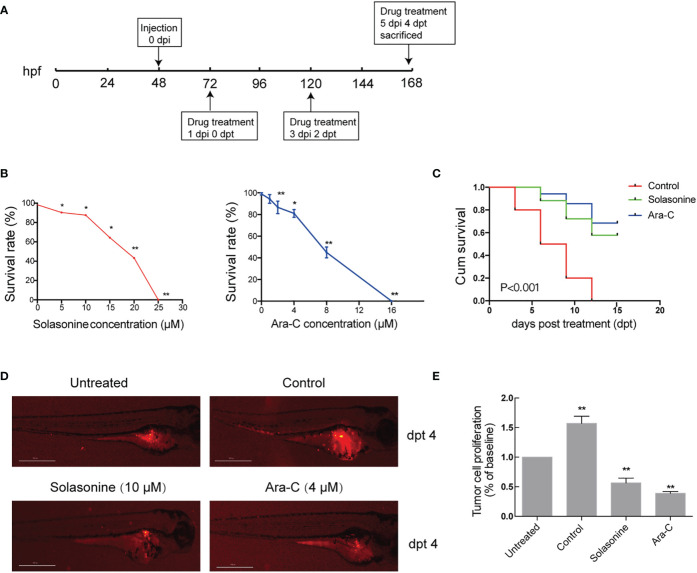
Solasonine inhibited tumor growth and metastasis in the zebrafish xenograft model. **(A)** A Schematic diagram of the timeline for cell injection and drug treatment. **(B)** The maximum safe dose was 10 μM for solasonine and 4μM for Ara-C. **(C)** Solasonine and Ara-C prolonged the survival of zebrafish embryos compared to the control group. **(D)** Solasonine and Ara-C lowered the fluorescence intensity compared with the control group at dpt 4. Untreated means zebrafish xenograf untreated with drugs at 0 dpt. **(E)** Bar chart showed the tumor cell proliferation treated by solasonine and Ara-C at 4 dpt. (dpt represented days post treatment, hpf represented hours post fertilization, dpi represented hours post injection. * considered a statistical difference compared to the untreated with drugs, *P < 0.05, **P < 0.01).

The antineoplastic activities of solasonine were also evaluated in a zebrafish xenograft model using the human acute monocytic leukemic cell line THP-1. A schematic diagram of the timeline for cell injection and drug treatment is shown in **Figure 7A**. At 72 hpf, various concentrations of solasonine (0, 5, 10, 15, 20, or 25 μM) and Ara-C (0, 1, 2, 4, 8, or 16 μM) were administered to the zebrafish embryos *via* soaking for 4 days. The results showed that the maximum safe dose was 10 μM for solasonine and 4μM for Ara-C (**Figure 7B**). To evaluate the growth and metastasis of the tumor xenograft, we observed the zebrafish xenograft at 4 dpt through an inverted fluorescent microscope. The results showed that solasonine and Ara-C lowered the fluorescence intensity compared with the control group at 4 dpt. Meanwhile, the metastasis to the embryos tails and body also decreased (**Figure 7D**). The cell number at 0 dpt was set as the baseline and was normalized to 1. The THP-1 cells proliferated by 1.57-fold at 4 dpt in the control group, but proliferation decreased by 0.56- and 0.39-fold in the 10 μM solasonine treatment group and in the 4 μM Ara-C group at 4 dpt, respectively (**Figure 7E**). At the same time, we observed that solasonine prolonged the survival of zebrafish embryos compared to the control group (**Figure 7C**). These results indicated that solasonine was actively inhibited tumor growth and metastasis in a time-dependent manner in the zebrafish xenograft model.

## Discussion

Herbal plants and both their semi-synthetic and synthetic derivatives have drawn increasing attention due to their antitumor effects (26). Paclitaxel, doxorubicin, and vincristine are significant anticancer compounds derived from nature and represent milestone chemotherapeutic agents (27). Solasonine is a steroidal alkaloid which is mainly found in *Solanum nigrum L*. Solasonine reportedly suppressed the growth of several tumor cells, especially of solid tumors such as, glioma (28), colon carcinoma (15), cervical adenocarcinoma (15), hepatocellular liver carcinoma (15), breast cancer (29), and lung cancer (30). Our study revealed that solasonine exerted antiproliferative activity against the acute monocytic leukemic cell lines THP-1 and MV4-11, in a dose- and time-dependent manner. We also observed that solasonine could inhibited tumor growth in a xenograft model. These results confirmed that the solasonine exerts anti-acute monocytic leukemia effects both *in vivo* and *in vitro*. Further analysis indicated that solasonine also inhibited the progression of acute monocytic leukemia by promoting apoptosis and inducing cell cycle arrest.

Apoptosis has been recognized as a unique and significant pattern of “programmed” cell death, which involves the removal of genetically determined cells. It is characterized by specific morphological changes in cell structure and enzyme-dependent biochemical processes. The apoptosis pathway generally includes an initial reception of an apoptosis signal, which activates initiator caspases (caspases 8 and 9), which then leads to the activation of executioner caspases (caspases 3, 6, and 7), eventually, resulting in DNA fragmentation (31, 32). Several studies revealed that solasonine regulates proliferation by promoting apoptosis in other cancers, such as in the lung cancer cell line, as described by Huang et al. (30). Solasonine also induced human cholangiocarcinoma epithelial QBC939 cells apoptosis (33). However, studies on acute monocytic leukemia are rare. In this study, we concluded that solasonine promoted apoptosis on the acute monocytic leukemia cell lines THP-1 and MV4-11 by flow cytometry analysis, western blotting analysis, and the TUNEL assay. These results indicated that solasonine induced apoptosis of acute monocytic leukemia cells.

The regulation of the cell cycle can precisely control DNA replication, cell division, and other important events to maintain DNA integrity (34). DNA damage can lead to G1 or G2 phase cell cycle arrest or delay (35). Two checkpoints of G1/S and G2/M phase are important in the cell cycle process, and the combination and activity gain and loss of CDK-cyclin play a key role (36). In the G2/M phase, CDK1/cyclinB1 determines the fate of the cell cycle. For various human cancers, dysregulation of the cell cycle is an important course of tumorigenesis and progression (37). Jin et al. observed that solasonine induced a higher proportion of human HCC cells to accumulate in the G2/M phase, which means solasonine caused cell cycle arrest in the G2/M phase (38). However, other studies have indicated that solasonine caused cell cycle arrest in the S phase, such as those by Wang et al. (28) and Fekry et al. (39). In our study, we found that exposure to solasonine upregulated the expression of P-CDK1 and downregulated cyclin B1 expression in THP-1 and MV4-11 cell lines through immunoblotting analysis. Subsequently, flow cytometry studies indicated that solasonine increased the proportion of cells in the G2/M phase. Overall, our findings indicated that solasonine induced the cell cycle arrest of acute monocytic leukemic cell lines in the G2/M phase.

FOX is downstream of several kinases, such as AKT, JNK, and AMPK. AKT directly phosphorylates FOXOs and allows its binding to 14-3-3 nuclear output proteins, and translocation of FOXOs from the nucleus to the cytoplasm, where its accumulation leads to subsequent degradation (40). In addition, other kinases associated with carcinogenic signals, such as SGK, CK1, and IKK, can inactivate FOXO by a similar mechanism. However, AMPK can phosphorylate FOXO3A at six different threonine and serine sites to activate its transcriptional activity and regulate the transcription of downstream genes (41). In mesenchymal stem cells, AMPK can phosphorylate FOXO3A, and phosphorylated FOXO3A enters the nucleus to enhance transcriptional activity (42). In our immunofluorescence staining assay and immunoblotting analysis, we found that solasonine promoted FOXO3A nuclear translocation. However, there are no similar studies evaluating solasonine effects on AMPK/FOXO3A signaling. Our results showed that treatment with solasonine increased the expression of P-AMPK and induced the nuclear translocation of FOXO3A in THP-1 and MV4-11 cell lines, indicating the ability of solasonine to active AMPK and FOXO3A. Further, down-regulation of AMPK activity was achieved by compound C when co-treated with solasonine, whereby we also observed reduced nuclear translocation of FOXO3A, apoptosis, and cell cycle arrest.

## Conclusions

**Figure 8:**
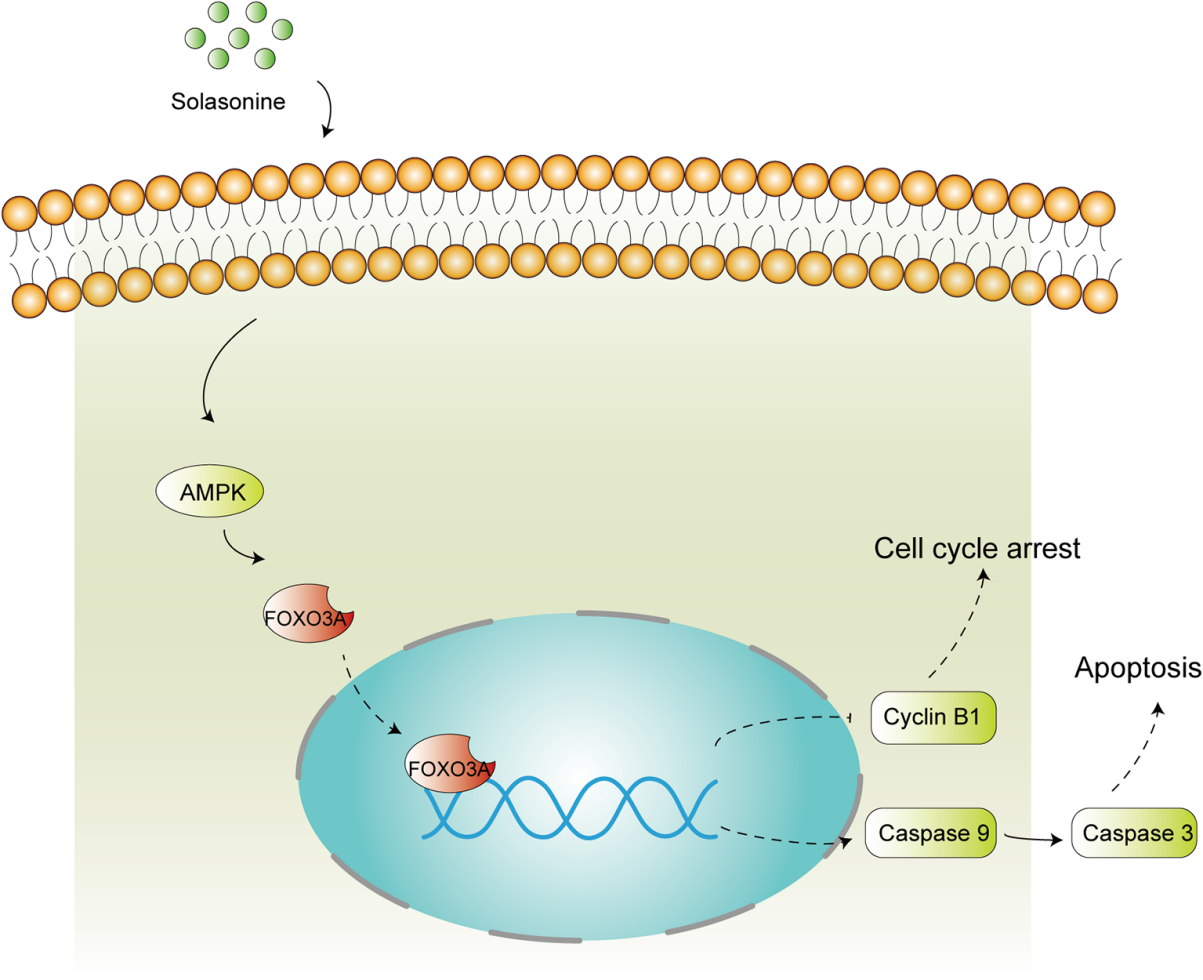
Schematic diagram of solasonine induced cell apoptosis and cell cycle arrest through AMPK/FOXO3A axis. Solasonine activating AMPK and then inducing FOXO3A nuclear translocation and finally exerting two aspect function of cell cycle arrest and apoptosis through inhibiting the expression of Cyclin B1 and promoting the expression of the caspase9, caspase3.

In summary, the present study reported the beneficial effects of solasonine against the progression of acute monocytic leukemia and we also identified the underlying molecular mechanisms of action. Overall, solasonine inhibits the progression of acute monocytic leukemia by promoting apoptosis and inducing cell cycle arrest, and the underlying mechanism correlated with high expression of the AMPK/FOXO3A pathway (**Figure 8**).

## Data Availability Statement

The original contributions presented in the study are publicly available. This data can be found here: https://www.ncbi.nlm.nih.gov/sra; PRJNA669277.

## Ethics Statement

Animal experiments were approved by the Animal Ethics committee in Affiliated Hospital of Nanjing University of Traditional Chinese Medicine.

## Author Contributions

HZ and FT designed, performed, and wrote article. PJ performed the flow cytometry analysis and analyzed the data. XD, BM, and MW conducted the animal study. HD and XCS conducted the partial immunoblotting analysis. XZ and ZY analyzed Next-Gen RNA Sequencing and the relationship between solasonine and AMPK/FOXO3A. XMS design and revised the study. All authors contributed to the article and approved the submitted version.

## Funding

This study was supported by the National Natural Science Foundation of China (No. 8157140609), Research and Innovation Plan for Graduate Students for Jiangsu Province in 2020 (No. KYCX20_1474), and the Open Projects of the Discipline of Chinese Medicine of Nanjing University of Chinese Medicine Supported by the Subject of Academic priority discipline of Jiangsu Higher Education Institutions (ZYX03KF037 and ZYX03KF042).

## Conflict of Interest

The authors declare that the research was conducted in the absence of any commercial or financial relationships that could be construed as a potential conflict of interest.

## References

[1] Bennett JM Catovsky D Daniel MT Flandrin G Galton DA Gralnick HR Proposed revised criteria for the classification of acute myeloid leukemia. A report of the French American-British Cooperative Group. Ann Intern Med (1985) 103(4):620–5. 10.7326/0003-4819-103-4-620 3862359

[2] Porcu P Cripe LD Ng EW Bhatia S Danielson CM Orazi A Hyperleukocytic leukemias and leukostasis: a review of pathophysiology, clinical presentation and management. Leukemia Lymphoma (2000) 39(1-2):1–18. 10.3109/10428190009053534 10975379

[3] Peterson L Dehner LP Brunning RD Extramedullary masses as presenting features of acute monoblastic leukemia. Am J Clin Pathol (1981) 75(2):140–8. 10.1093/ajcp/75.2.140 6937136

[4] Mangal AK Grossman L Vickars L Disseminated intravascular coagulation in acute monoblastic leukemia: response to heparin therapy. Can Med Assoc J (1984) 130(6):731–3. PMC1875921 6582992

[5] Schoch C Schnittger S Klaus M Kern W Hiddemann W Haferlach T AML with 11q23/MLL abnormalities as defined by the WHO classification: incidence, partner chromosomes, FAB subtype, age distribution, and prognostic impact in an unselected series of 1897 cytogenetically analyzed AML cases. Blood (2003) 102(7):2395–402. 10.1182/blood-2003-02-0434 12805060

[6] Yan XJ Xu J Gu ZH Pan CM Lu G Shen Y Exome sequencing identifies somatic mutations of DNA methyltransferase gene DNMT3A in acute monocytic leukemia. Nat Genet (2011) 43(4):309–15. 10.1038/ng.788 21399634

[7] Boissel N Renneville A Biggio V Philippe N Thomas X Cayuela JM Prevalence, clinical profile, and prognosis of NPM mutations in AML with normal karyotype. Blood (2005) 106(10):3618–20. 10.1182/blood-2005-05-2174 16046528

[8] Wang CK Lin YF Tai CJ Wang CW Chang YJ Choong CY Integrated Treatment of Aqueous Extract of Solanum nigrum-Potentiated Cisplatin-and Doxorubicin-Induced Cytotoxicity in Human Hepatocellular Carcinoma Cells. Evidence-Based Complement Altern Med (2015) 545–59. 10.1155/2015/675270 PMC4499398 26221175

[9] Li J Li Q Feng T Zhang T Li K Zhao R Antitumor activity of crude polysaccharides isolated from Solanum nigrum Linne on U14 cervical carcinoma bearing mice. Phytother Res (2007) 21(9):832–40. 10.1002/ptr.2163 17486683

[10] Razali FN Sinniah SK Hussin H Zainal Abidin N Shuib AS Tumor suppression effect of Solanum nigrum polysaccharide fraction on Breast cancer via immunomodulation. Int J Biol Macromolecules (2016) 92:185–93. 10.1016/j.ijbiomac.2016.06.079 27365117

[11] Pan B Zhong W Deng Z Lai C Chu J Jiao G Inhibition of prostate cancer growth by solanine requires the suppression of cell cycle proteins and the activation of ROS/P38 signaling pathway. Cancer Med (2016) 5(11):3214–22. 10.1002/cam4.916 PMC5119977 27726305

[12] Tai CJ Wang CK Tai CJ Lin YF Lin CS Jian JY Aqueous Extract of Solanum nigrum Leaves Induces Autophagy and Enhances Cytotoxicity of Cisplatin, Doxorubicin, Docetaxel, and 5-Fluorouracil in Human Colorectal Carcinoma Cells. Evidence-Based Complement Altern Med (2013) 2013:e514719. 10.1155/2013/514719 PMC3703357 23843876

[13] Tai CJ Choong CY Shi YC Lin YC Wang CW Lee BH Solanum nigrum Protects against Hepatic Fibrosis via Suppression of Hyperglycemia in High-Fat/Ethanol Diet-Induced Rats. Molecules (Basel Switzerland) (2016) 21(3):269. 10.3390/molecules21030269 PMC6274119 26927042

[14] Liu FP Ma X Li MM Li Z Han Q Li R Hepatoprotective effects of Solanum nigrum against ethanol-induced injury in primary hepatocytes and mice with analysis of glutathione S-transferase A1. J Chin Med Assoc (2016) 79(2):65–71. 10.1016/j.jcma.2015.08.013 26775601

[15] Munari CC de Oliveira PF Campos JC Martins Sde P Da Costa JC Bastos JK Antiproliferative activity of Solanum lycocarpum alkaloidic extract and their constituents, solamargine and solasonine, in tumor cell lines. J Natural Medicines (2014) 68(1):236–41. 10.1007/s11418-013-0757-0 23475509

[16] Yubing J Shenghui W Shiyong G Chenfeng J Xiang J Effect of Solanum nigrum alkaloid on membrane fluidity and membrane protein level of tumor cells in H22 tumor-bearing mice. Chin Tradit Herbal Drugs (2005) 02):239–41.

[17] Yubing J Hongliang W Shiyong G Effect of solanine on DNA and RNA in tumor cell of tumor-bearing mice. Chin Tradit Herbal Drugs (2005) 08):84–6.

[18] Nho RS Hergert P FoxO3a and disease progression. World J Biol Chem (2014) 5(3):346–54. 10.4331/wjbc.v5.i3.346 PMC4160528 25225602

[19] Vogt PK Jiang H Aoki M Triple layer control: phosphorylation, acetylation and ubiquitination of FOXO proteins. Cell Cycle (Georgetown Tex) (2005) 4(7):908–13. 10.4161/cc.4.7.1796 15917664

[20] Shackelford DB Shaw RJ The LKB1-AMPK pathway: metabolism and growth control in tumour suppression. Nat Rev Cancer (2009) 9(8):563–75. 10.1038/nrc2676 PMC2756045 19629071

[21] Chiacchiera F Simone C The AMPK-FoxO3A axis as a target for cancer treatment. Cell Cycle (Georgetown Tex) (2010) 9(6):1091–6. 10.4161/cc.9.6.11035 20190568

[22] Queiroz EA Puukila S Eichler R Sampaio SC Forsyth HL Lees SJ Metformin induces apoptosis and cell cycle arrest mediated by oxidative stress, AMPK and FOXO3a in MCF-7 breast cancer cells. PloS One (2014) 9(5):e98207. 10.1371/journal.pone.0098207 24858012 PMC4032293

[23] Shrestha A Nepal S Kim MJ Chang JH Kim SH Jeong GS Critical Role of AMPK/FoxO3A Axis in Globular Adiponectin-Induced Cell Cycle Arrest and Apoptosis in Cancer Cells. J Cell Physiol (2016) 231(2):357–69. 10.1002/jcp.25080 26089158

[24] Liu Z Ma C Tang X Tang Q Lou L Yu Y The Reciprocal Interaction Between LncRNA CCAT1 and miR-375-3p Contribute to the Downregulation of IRF5 Gene Expression by Solasonine in HepG2 Human Hepatocellular Carcinoma Cells. Front Oncol (2019) 9:1081. 10.3389/fonc.2019.01081 31681610 PMC6813207

[25] Zhong Y Li S Chen L Liu Z Luo X Xu P In Vivo Toxicity of Solasonine and Its Effects on cyp450 Family Gene Expression in the Livers of Male Mice from Four Strains. Toxins (2018) 10(12):487. 10.3390/toxins10120487 PMC6315709 30477109

[26] Cragg GM Newman DJ Antineoplastic agents from natural sources: achievements and future directions. Expert Opin Invest Drugs (2000) 9(12):2783–97. 10.1517/13543784.9.12.2783 11093353

[27] Mann J Natural products in cancer chemotherapy: past, present and future. Nat Rev Cancer (2002) 2(2):143–8. 10.1038/nrc723 12635177

[28] Wang X Zou S Lan YL Xing JS Lan XQ Zhang B Solasonine inhibits glioma growth through anti-inflammatory pathways. Am J Trans Res (2017) 9(9):3977–89. PMC5622243 28979674

[29] Akter R Uddin SJ Tiralongo J Grice ID Tiralongo E A New Cytotoxic Steroidal Glycoalkaloid from the Methanol Extract of Blumea lacera Leaves. J Pharm Pharm Sci: Publ Can Soc Pharm Sci Soc Can Des Sci Pharm (2015) 18(4):616–33. 10.18433/J3161Q 26626252

[30] Huang W Wang Y Zhu H Wu Y Xie X Wang D Solasonine-induced Apoptosis in Lung Cancer Cell Line H446 and Its Mechanism. Chin J Lung Cancer (2015) 18(7):416–21. 10.3779/j.issn.1009-3419.2015.07.05 PMC6000244 26182866

[31] Martinvalet D Zhu P Lieberman J Granzyme A induces caspase-independent mitochondrial damage, a required first step for apoptosis. Immunity (2005) 22(3):355–70. 10.1016/j.immuni.2005.02.004 15780992

[32] Elmore S Apoptosis: a review of programmed cell death. Toxicologic Pathol (2007) 35(4):495–516. 10.1080/01926230701320337 PMC2117903 17562483

[33] Zhanpeng Y Xiaosong W Tingting X Zhentao A Xiuhua Z Fangshi Z Apoptosis-inducing Effects of Solasonine on Human Cholangiocarcinoma Epithelial QBC939 Cells and Its Mechanism. Natural Product Res Dev (2017) 29(04):559–62. 10.16333/j.1001-6880.2017.4.004

[34] Sancar A Lindsey-Boltz LA Unsal-Kaçmaz K Linn S Molecular mechanisms of mammalian DNA repair and the DNA damage checkpoints. Annu Rev Biochem (2004) 73:39–85. 10.1146/annurev.biochem.73.011303.073723 15189136

[35] Musacchio A Salmon ED The spindle-assembly checkpoint in space and time. Nat Rev Mol Cell Biol (2007) 8(5):379–93. 10.1038/nrm2163 17426725

[36] Lim S Kaldis P Cdks, cyclins and CKIs: roles beyond cell cycle regulation. Development (2013) 140(15):3079–93. 10.1242/dev.091744 23861057

[37] Bisteau X Caldez MJ Kaldis P The Complex Relationship between Liver Cancer and the Cell Cycle: A Story of Multiple Regulations. Cancers (Basel) (2014) 6(1):79–111. 10.3390/cancers6010079 24419005 PMC3980619

[38] Jin M Shi C Li T Wu Y Hu C Huang G Solasonine promotes ferroptosis of hepatoma carcinoma cells via glutathione peroxidase 4-induced destruction of the glutathione redox system. BioMed Pharmacother (2020) 129:110282. 10.1016/j.biopha.2020.110282 32531676

[39] Fekry MI Ezzat SM Salama MM Alshehri OY Al-Abd AA-O Bioactive glycoalkaloides isolated from Solanum melongena fruit peels with potential anticancer properties against hepatocellular carcinoma cells. Sci Rep (2019) 9(1):1746. 10.1038/s41598-018-36089-6 30741973 PMC6370831

[40] Calnan DR Brunet A The FoxO code. Oncogene (2008) 27(16):2276–88. 10.1038/onc.2008.21 18391970

[41] Greer EL Oskoui PR Banko MR Maniar JM Gygi MP Gygi SP The energy sensor AMP-activated protein kinase directly regulates the mammalian FOXO3 transcription factor. J Biol Chem (2007) 282(41):30107–19. 10.1074/jbc.M705325200 17711846

[42] Yang M Pi H Li M Xu S Zhang L Xie J From the Cover: Autophagy Induction Contributes to Cadmium Toxicity in Mesenchymal Stem Cells via AMPK/FOXO3a/BECN1 Signaling. Toxicol Sci (2016) 154(1):101–14. 10.1093/toxsci/kfw144 27492225

