# Supplementary material for: Solasonine Suppresses the Proliferation of Acute Monocytic Leukemia Through the Activation of the AMPK/FOXO3A Axis
Source: Front Oncol. 2021 Jan 29;10:614067. doi: 10.3389/fonc.2020.614067 (PMC7879981; doi:10.3389/fonc.2020.614067)
Supplement: Supplementary file 1 [file DataSheet_1.docx]

***Supplementary Materials***


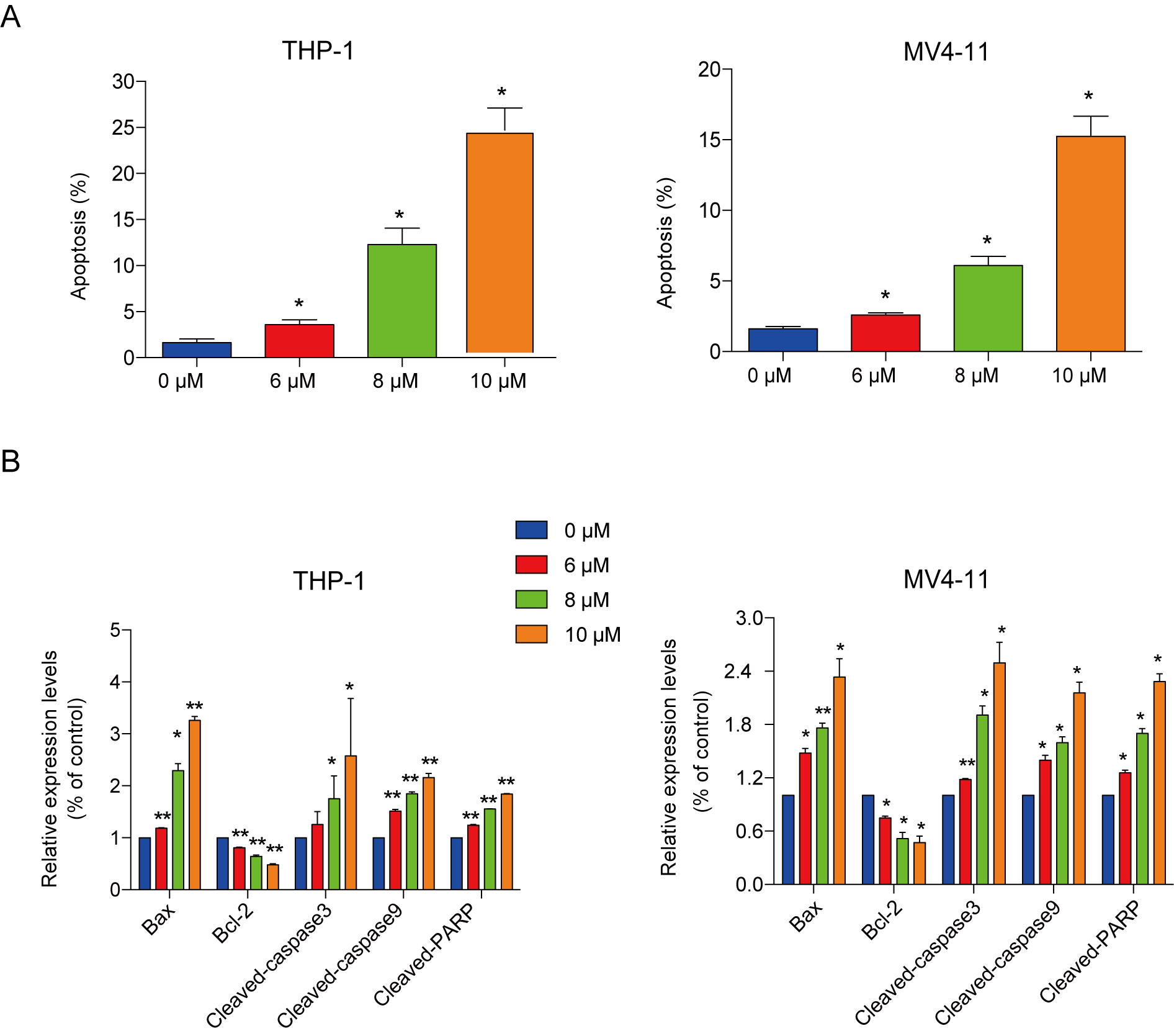


**Supplementary Figure 1|** Solasonine promoted acute monocytic leukemic cells apoptosis. **(A)** Bar chart showed the apoptosis rate induced by solasonine through the flow cytometry analysis **(B)** Bar chart showed the apoptosis relative protein expression. (*considered a statistical difference compared to the control group, *P < 0.05, **P < 0.01)


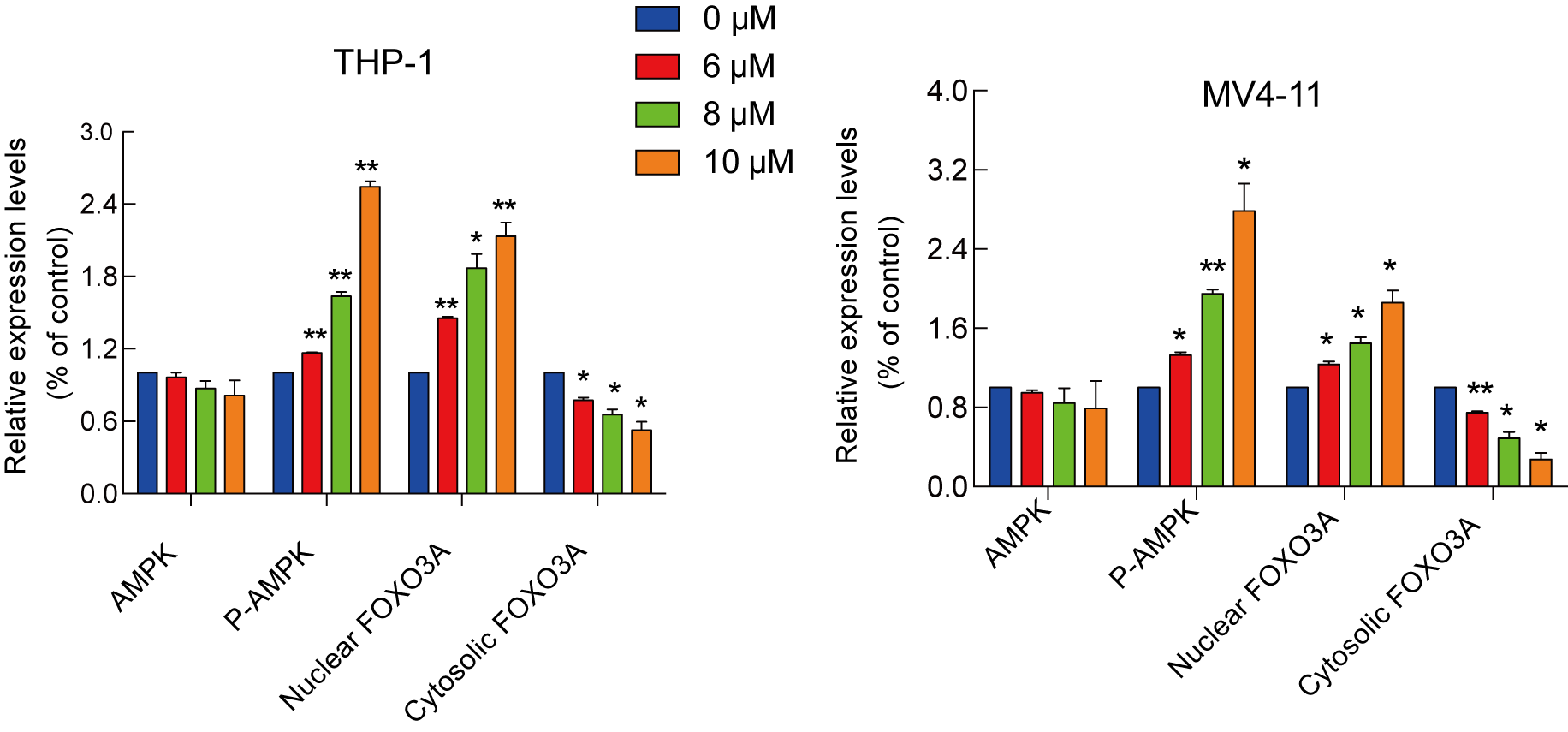


**Supplementary Figure 2|** Solasonine activated AMPK/FOXO3A axis. Bar chart showed that solasonine upregulated the expression of P-AMPK and caused nuclear translocation of FOXO3A by immunoblotting analysis. (*considered a statistical difference compared to the control group, *P < 0.05, **P < 0.01)


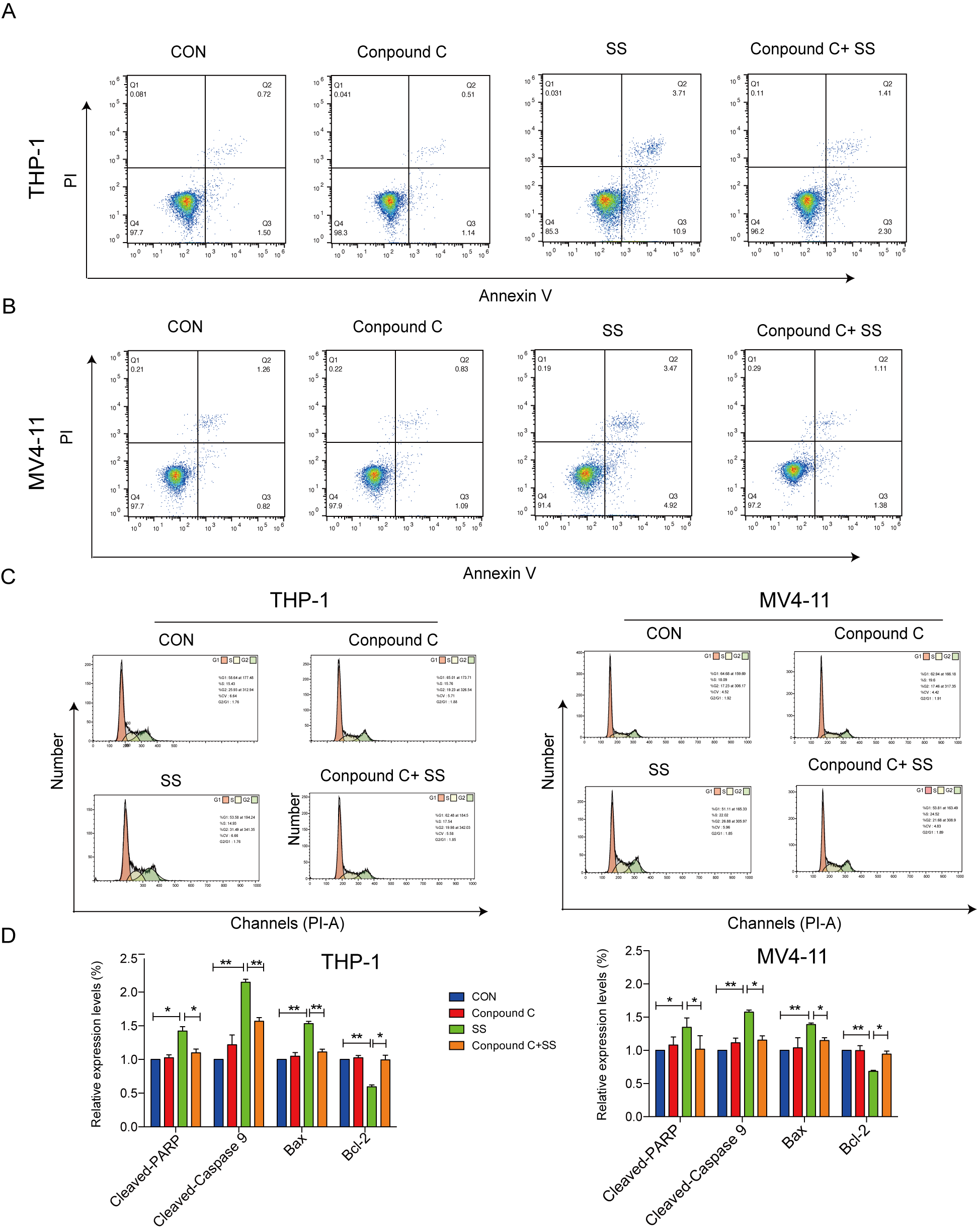


**Supplementary Figure 3|** AMPK/FOXO3A signaling was required for solasonine-induced cell cycle arrest and apoptosis in acute monocytic leukemic cell lines. **(A-B)** Flow cytometry analysis showed compound C decreased the solasonine induced apoptosis. **(C)** Flow cytometry analysis showed compound C restored the cell cycle arrest in the G2/M phase. **(D)** Bar chart showed that compound C decreased the solasonine-induced apoptosis related protein in THP-1 and MV4-11cells. (*considered a statistical difference compared to the control group, *P < 0.05, **P < 0.01)
